# Steroid Induced Central Serous Chorioretinopathy in Giant Cell Arteritis

**DOI:** 10.1155/2013/924037

**Published:** 2013-06-11

**Authors:** Andre Grixti, Vineeth Kumar

**Affiliations:** ^1^Department of Ophthalmology, Arrowe Park Hospital, Arrowe Park Road, Upton, Wirral CH49 5PE, UK; ^2^Department of Ophthalmology, Royal Liverpool University Hospital, Prescot Street, Liverpool L7 8XP, UK

## Abstract

Giant cell arteritis (GCA) is an ophthalmic emergency which requires early diagnosis and treatment with high dose systemic corticosteroids in order to prevent permanent visual loss. However, systemic corticosteroids have significant ocular side effects including cataract formation, raised intraocular pressure, and less commonly, central serous chorioretinopathy (CSCR). We report a case of visual loss secondary to CSCR complicating corticosteroid therapy in GCA. When assessing patients with systemic conditions such as GCA or other vasculitic process, who complain of visual loss which is getting worse on corticosteroid treatment, clinicians should consider other causes such as CSCR as part of the differential diagnosis. Extra caution should be exercised in such cases as increasing the dose of corticosteroids might aggravate CSCR resulting in further visual loss.

## 1. Introduction

Giant cell arteritis (GCA) is an ophthalmic emergency which requires urgent treatment with systemic corticosteroids to prevent blindness. Visual impairment secondary to GCA is usually attributed to anterior ischaemic optic neuropathy or less commonly retinal vessel occlusion. Rarely, anterior segment ischaemia or choroidal infarction may also occur. However, corticosteroids have ocular side effects including cataract, raised intraocular pressure, and central serous chorioretinopathy (CSCR). We report a case of steroid induced CSCR in GCA. Only three cases like this have been previously described in the literature [1–3]. 

## 2. Methods

A retrospective case report format is used.

## 3. Results

A 67-year-old female with previous biopsy positive GCA presented with scalp tenderness, jaw claudication, and chest pain of one month duration. She had no visual symptoms. Erythrocyte sedimentation rate was 60 mm/hr, and C reactive protein was 22 mg/dL. CT angiogram of the aorta was normal. MRI brain revealed periventricular, and subcortical white matter changes suspicious of vasculitis. Intravenous methylprednisolone (1 g/day) was administered for 3 consecutive days followed by 60 mg oral prednisolone daily. Five days after initiating treatment, she complained of left central visual loss.

**Figure 1:**
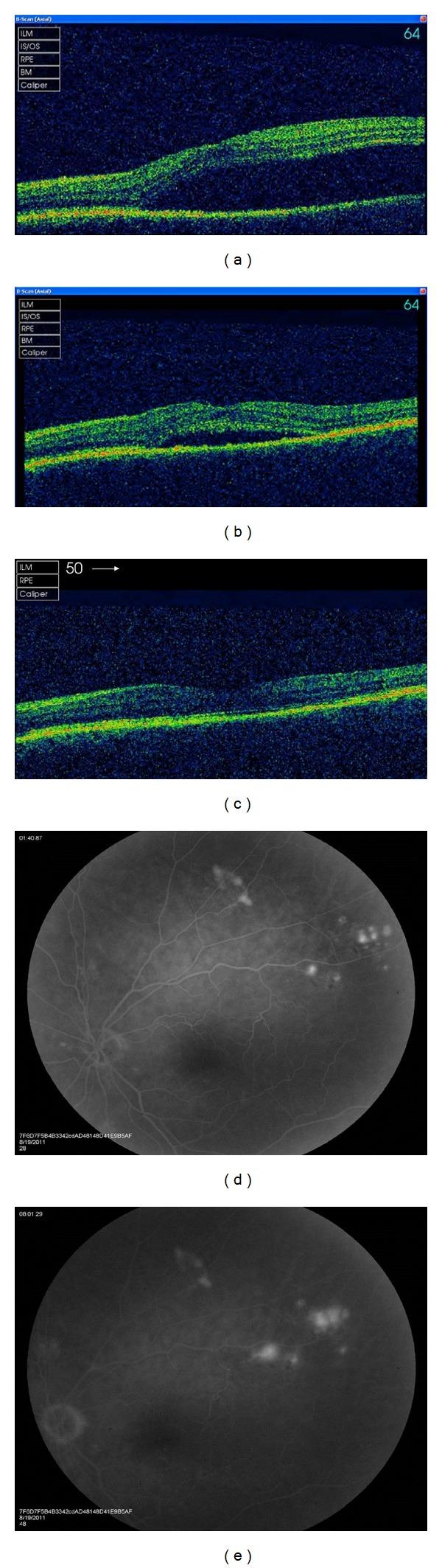
((a), (b), (c)) Optical coherence tomography of the left macula at presentation, 6 weeks and 6 months showing complete resolution of subretinal fluid with tapering of corticosteroids. ((d), (e)) Fundus fluorescein angiogram of the left eye showing multiple areas of focal pinpoint hyperfluorescence with progressive leakage in the late phase.

Examination revealed best corrected Snellen visual acuities of 6/6 right and 6/18 left, normal optic discs, and left serous macular detachment. CSCR was diagnosed by optical coherence tomography (OCT) and fundus fluorescein angiography (FFA). Oral prednisolone was gradually tapered off, and cyclophosphamide was added as a steroid sparing agent. At six weeks left visual acuity improved to 6/9, and subretinal fluid reduced and completely resolved at 6 months (Figure 1).

## 4. Discussion

CSCR is an idiopathic disorder characterised by serous detachment of the neurosensory retina from the retinal pigment epithelium (RPE) at the macula, secondary to focal RPE defects. CSCR secondary to corticosteroids is well documented in the literature [4–6]; however, only few reports identified this condition during treatment of GCA [1, 2]. 

Corticosteroids disrupt RPE tight junctions which constitute the outer blood retinal barrier, leading to accumulation of subretinal fluid [2]. Increased choriocapillary fragility, hyperpermeability, and ion pumping dysfunction have been implicated [1]. 

When assessing patients with GCA or vasculitis who complain of visual loss getting worse on corticosteroids, clinicians should consider CSCR as part of the differential diagnosis [1, 2]. In such cases discontinuation of corticosteroids may be contraindicated. Close monitoring of clinical signs and inflammatory markers and use of steroid sparing medication such as cyclophosphamide permit more rapid tapering of corticosteroids [1]. This was particularly relevant in our case as it allowed reduction in the daily dosage of corticosteroids while keeping vasculitis under control.
